# Improving the Efficiency of Fan Coil Units in Hotel Buildings through Deep-Learning-Based Fault Detection

**DOI:** 10.3390/s23156717

**Published:** 2023-07-27

**Authors:** Iva Matetić, Ivan Štajduhar, Igor Wolf, Sandi Ljubic

**Affiliations:** 1 Faculty of Engineering, University of Rijeka, Vukovarska 58, HR-51000 Rijeka, Croatia; iva.matetic@riteh.hr (I.M.); ivan.stajduhar@riteh.hr (I.Š.); igor.wolf@riteh.hr (I.W.); 2 Center for Artificial Intelligence and Cybersecurity, University of Rijeka, R. Matejcic 2, HR-51000 Rijeka, Croatia

**Keywords:** fan coil units, HVAC systems, fault detection, deep learning, CNN, LSTM, GRU, RF

## Abstract

Optimizing the performance of heating, ventilation, and air-conditioning (HVAC) systems is critical in today’s energy-conscious world. Fan coil units (FCUs) play a critical role in providing comfort in various environments as an important component of HVAC systems. However, FCUs often experience failures that affect their efficiency and increase their energy consumption. In this context, deep learning (DL)-based fault detection offers a promising solution. By detecting faults early and preventing system failures, the efficiency of FCUs can be improved. This paper explores DL models as fault detectors for FCUs to enable smarter and more energy-efficient hotel buildings. We tested three contemporary DL modeling approaches: convolutional neural network (CNN), long short-term memory network (LSTM), and a combination of CNN and gated recurrent unit (GRU). The random forest model (RF) was additionally developed as a baseline benchmark. The fault detectors were tested on a real-world dataset obtained from the sensory measurement system installed in a hotel and additionally supplemented with simulated data via a physical model developed in TRNSYS. Three representative FCU faults, namely, a stuck valve, a reduction in airflow, and an FCU outage, were simulated with a much larger dataset than is typically utilized in similar studies. The results showed that the hybrid model, integrating CNN and GRU, performed best for all three observed faults. DL-based fault detectors outperformed the baseline RF model, confirming these solutions as viable components for energy-efficient hotels.

## 1. Introduction

Fan coil units (FCUs) play a crucial role in public buildings such as hotels, contributing to occupants’ comfort and overall energy consumption. They work by circulating air through a heat exchanger coil that is either heated or cooled using a working fluid. Like any mechanical system, FCUs are prone to defects and malfunctions that can reduce their efficiency and increase energy costs. These inefficiencies, if not corrected, can have a negative impact on the environment over time. Although this paper is primarily concerned with fault detection in FCUs, it is important to recognize that they are part of the broader category of heating, ventilation, and air-conditioning (HVAC) systems. Therefore, in the following sections, we look at the challenges and issues associated with HVAC systems as a whole.

The impact of HVAC systems goes beyond energy consumption, as people spend more than 80% of their time indoors, and therefore the health and well-being of occupants can be affected by indoor air quality [1]. Buildings are responsible for about one-third of the world’s energy consumption and one-quarter of global CO${}_{2}$ emissions. In some high-energy-consuming countries, their contribution to energy consumption is even more significant, reaching 42% in Russia, 41% in the EU, 37% in Japan, and 34% in the USA [2]. About 5% of buildings in the European Union are older than 50 years, while almost 75% of the existing building stock is energy-inefficient [3]. A similar trend of old inefficient houses is also observed on other continents and in countries such as the USA, where houses are between 20 and 31 years old on average [4]. The link between aging homes and energy efficiency is evident from various factors such as outdated HVAC systems, inadequate insulation, faulty window seals and door openings, and obsolete appliances [5].

Tourism is responsible for greenhouse gas emissions, with $CO_{2}$ accounting for the majority of total emissions (3.2 billion tons of $CO_{2}$) at 72%, mainly from transportation, electricity consumption in hotels and restaurants, and the burning of fossil fuels in the production of goods purchased by tourists [6]. By 2030, the global electricity consumption for cooling buildings is expected to increase by up to 40%. Heat pumps, a key component of HVAC systems, are expected to account for 20% of global heating demand in buildings by 2030. These targets underscore the urgency of adopting sustainable practices in HVAC systems to reduce $CO_{2}$ emissions and meet climate targets set by the International Energy Agency (IEA). The IEA’s net-zero emissions plan calls for a 40% reduction in greenhouse gas emissions from buildings between 2020 and 2030, requiring a shift away from fossil fuel boilers and improved energy efficiency in existing buildings [7].

To minimize economic risk and promote long-term sustainable operations, low-carbon design, resource-optimized management, and smart hotel service systems have demonstrated their potential to reduce energy consumption and carbon emissions. Green hotel practices, such as energy-saving measures and waste-reduction programs, have been introduced to mitigate the negative environmental impact of the hotel industry [8].

This paper is about integrating a deep learning (DL) model into HVAC systems to make buildings, especially hotels, smarter and more energy-efficient. By addressing the environmental challenges of HVAC systems and leveraging the potential of DL, hotels can play an important role in promoting sustainability, reducing energy consumption, and mitigating the environmental impacts associated with the hospitality industry.

### 1.1. Related Work

Fault detection and diagnosis (FDD) methods in HVAC systems use various techniques to detect anomalies and failures of system components. By analyzing sensor readings and performance data using data analytics, machine learning (ML) algorithms, and signal processing, FDD methods can continuously monitor the system and classify faults. The application of FDD methods in HVAC systems increases user comfort, reduces maintenance costs, and extends equipment life. Thus, the early detection of faults leads to both a higher energy efficiency of the system and indirectly improves the efficiency of the system components, of which FCUs are the focus of this paper. Integrating FDD methods into HVAC systems is therefore an important step towards achieving energy efficiency and sustainability goals, promoting resource conservation, and creating a greener and more environmentally responsible future.

Recent advances in FDD methods encompass a range of approaches, including rule-based methods, statistical analysis, pattern recognition, and DL and ML algorithms, which aim to improve the accuracy and automation of the FDD process. Among these approaches, the data-driven methodology, especially using ML or DL algorithms, has gained considerable recognition in the field of HVAC systems [9]. Data-driven techniques offer practical advantages as they can be implemented without extensive knowledge of the building or expertise in energetics and thermodynamics. Notable examples of ML techniques include the use of random forest (RF) models, which have been proven to be the primary classifier compared to k-nearest neighbors (KNN), support vector machines (SVMs), and decision trees (DTs) [10,11]. RF models are also suitable for feature extraction and can be combined with robust classifiers such as SVMs [12,13]. SVMs have demonstrated proficiency as classifiers, especially when dealing with datasets with a small number of features. Notable implementations include binary classifiers and multiclass solutions [14,15]. Multilayer perceptrons (MLPs) and decision trees are both widely used models in this area. DTs are known for their high interpretability, which makes them valuable for interpreting models beyond MLPs [16]. Moreover, MLPs have been used as self-training MLPs in previous studies [17,18].

Besides the aforementioned traditional ML models, convolutional neural networks (CNNs) and recurrent neural networks (RNNs) have emerged as extremely popular alternatives in the field of DL. CNNs are mainly used in computer vision tasks and utilize their ability to analyze and recognize visual input. RNNs, on the other hand, are designed to process sequential data effectively and use their ability to process information in a temporal manner, similar to how CNNs process spatial structures in image inputs [19]. The application of DL techniques in the industry for fault detection began in 2013. Over the years, from 2016 to the present, CNNs have been the most widely used, followed by RNNs, generative adversarial networks (GANs), and autoencoders (AE) as prominent alternatives [20]. The emergence of DL implementations in HVAC system research can be traced back to 2018, with the seminal paper by Guo et al. [21] on using a deep belief network (DBN) for fault diagnosis in air-conditioning systems. This study laid the foundation for subsequent research in this field [22].

Several notable models have been proposed for 1D CNNs in HVAC, including work by Li et al. [23], Liao et al. [24], and Cheng et al. [25]. Similarly, Liu et al. [26] and Fan et al. [27] presented new approaches for 2D CNNs. Hence, CNNs have been shown to be versatile in handling HVAC data, either in 1D or 2D form. Although both 1D and 2D representations are well described in the literature, 1D forms are generally preferred due to their simpler preprocessing requirements, unlike 2D CNNs, which usually require data conversion to images [22].

Moreover, recurrent networks are essential for analyzing time series data, as they excel in capturing sequential dependencies, processing variable-length sequences, retaining historical information, automatically extracting relevant features, and performing tasks such as prediction and anomaly detection. In this context, recurrent models incorporating long short-term memory network (LSTM) architectures have been studied in detail by Liu et al. [28], Tian et al. [29], Taheri et al. [30], and Behravan et al. [31]. In addition, gated recurrent unit (GRU) models, a special type of RNN, have been proposed and used by Wang et al. [32] and Li et al. [33] in their respective studies.

### 1.2. Contributions and Structure

In the HVAC-related literature, researchers have focused on examining specific subcategories such as air handling units, air conditioners, heat pumps, and chillers. FCUs, however, have received relatively little attention and account for only about 6% of the research effort [9]. Therefore, we aim to fill the detected research gap and evaluate established DL architectures using real-world FCU data.

Researchers have presented good models and results in the selected related work [24,31,32,34]. They have generally done multiple classifications of different faults ranging from 4 to 10 different types of faults. The datasets in the respective studies are typically based on measurements with a low-frequency interval between consecutive samples, ranging from 1 s to 5 min. However, it is worth noting that the total volume of data in those datasets is considerably smaller than the dataset we utilized. The largest dataset commonly used is that of the American Society of Heating, Refrigerating, and Air-Conditioning Engineers (ASHRAE), which can include measurements of up to 1 year [32]. It is common for researchers to use data derived from the ASHRAE, or simulated data, as it is difficult to obtain labeled real-world data [9]. While we also used simulations to augment data, our approach differs significantly as we used an extensive real-world dataset spanning three years. We aimed to use this extensive dataset to improve the effectiveness of fault detection technologies based on DL methods, especially in the context of FCUs in HVAC systems.

Some authors have used a similar approach to collect real-world data and simulate faults [24,31,34]. However, as mentioned earlier, the volume of their datasets is much smaller and ranges from one day to only a few months. Our research used a unique dataset that includes three years of data from 60 hotel rooms where the corresponding sensor readings were collected at 5 min intervals. This dataset includes no less than 22 different features, which was also compared a lot to other studies. It contains real-world data, both sensor readings from the hotel and environmental information, as well as simulated faults generated by the corresponding TRNSYS model.

The contributions of this research are the following:

- The development of fault detection models for FCUs, an under-researched subtype of HVAC systems, to fill an existing gap in the literature and further advance the field of HVAC systems.
- The implementation of advanced DL-based architectures as the basis for fault detectors with the goal of optimizing the efficiency of FCUs in HVAC systems.
- The curation of an original dataset specific to FCUs focused on real data from a hotel building. This dataset served as the basis for developing four specialized models to identify three common faults in HVAC systems.
- The validation of the model architectures through rigorous testing on a large dataset and an analysis and comparison with a traditional RF-based model.

In the following, we present the underlying smart-room concept, which serves as the primary source of our data (Section 2.1). We provide detailed insights into the data-labeling procedure (Section 2.2), preprocessing techniques used (Section 2.3), and model development (Section 2.4). Results are presented and discussed in Section 3, after which we draw conclusions in Section 4.

## 2. Materials and Methods

In this section, we present the collected data used in our modeling pipeline. We also provide detailed information on the data preprocessing and model development, all of which were carried out using Python 3.9.13. Namely, while Pandas 1.5.1, Numpy 1.21.5, Matplotlib 3.5.2, and Scikit-learn 1.2.1 libraries were utilized for the data preprocessing and RF model development, the Pytorch 2.0.0 library was specifically used for DL model development.

### 2.1. Smart Room System and Data Acquisition

In this research, we utilized the same dataset as in our previous study [35]. The data were collected from a hotel building in Zagreb, the capital city of Croatia. The hotel employs a smart-room concept, which incorporates microprocessor-controlled stations for the efficient monitoring and regulation of crucial parameters to ensure the optimal functionality of the hotel rooms. These stations are connected to a central computer system, which enhances efficiency and provides comprehensive control. Consequently, this integrated system facilitates improved energy management, leading to potential cost savings, elevated customer service standards, enhanced system security, and increased operational efficiency through real-time access to current information. Implementing the smart-room concept empowers hotels to streamline their management activities, effectively oversee and monitor the entire system, and consequently achieve enhanced overall performance.

The acquired data spanned from 2015 to 2021, with a data sampling frequency of 5 min. However, we decided to focus on specific years, 2017 to 2019, for several reasons related to data quality and representativeness. We found that years 2017 to 2019 had the highest data quality in terms of reliability and accuracy. Unlike the other years in the dataset, which had numerous instances of missing data and recording errors, this time frame provided more consistent and trustworthy data. We also chose to exclude data from years 2020 and 2021 due to the significant impact of the pandemic on hotel operations. Due to the restrictions and limited travel during this time, fewer guests stayed at the hotel, which may have skewed the data and made it less representative of typical guest behavior and room temperatures. Therefore, to ensure the reliability, consistency, and relevance of our analysis and modeling, we selectively focused on data from 2017 to 2019 and specifically included three floors per year (upper and lower room temperatures from a single floor). It should be noted that this reduced dataset still contained many more data than the resources used in other studies.

Table 1 presents the data features. The environmental characteristics, such as outdoor temperature, humidity, and irradiation, were supplemented by the Croatian Meteorological and Hydrological Service. Moreover, we incorporated additional features such as room orientation and the status of the manually operated HVAC system through feature engineering.

### 2.2. Data Labeling

In order to label the data, we utilized a corresponding physical model. TRNSYS software was used to create a system model based on the architectural descriptions of the building. The model focused on simulating the thermal behavior of a specific part of the building, consisting of six thermal zones representing the guest rooms. By monitoring the central thermal zone in detail and creating boundary conditions in the other zones, the model ensured the consistency of physical properties in all rooms. To replicate the performance of the FCUs, performance maps were created with each operating point representing the output capacity under explicitly defined conditions. The operating points were obtained directly from the FCU manufacturer, and separate performance maps were created for heating and cooling modes. Specific input parameters were incorporated into the FCU model, such as the temperature and flow rate of water through the heat exchangers, the current characteristics of the room air, and the control system signals. The model then processed these inputs to obtain output variables that included the exact temperature, airflow, and relative humidity of the air entering the room. For detailed information on the development of the physical model, refer to our previous work [36], which offers comprehensive insights into the subject.

To ensure the accuracy of the simulations, the physical model was calibrated and validated against real data from the hotel building. The simulation environment used in the study was based on input features extracted from the hotel data mentioned in Table 1. These features were developed to control the FCU system and effectively regulate internal and external conditions. The thermostat control used the set temperature as input data to regulate the FCU. The Hvac_mode determined the heating or cooling demand in simulations, while the Hvac_state, initiated by the guest, interrupted the system’s operation. The HVAC_state_manual feature was used to set specific FCU speeds at specific times. Other parameters were used to simulate conditions in a hotel. Room_occupancy data were simulated as an internal heat source, and window-opening data were used to emulate the exchange of outdoor air with indoor air. To simulate the temperature conditions in the hotel, the temperatures in adjacent rooms were set as reference values from the collected data. Outdoor air temperature and solar irradiation were used to mimic external conditions. To ensure accuracy, the solar irradiation data were additionally processed to obtain accurate values for each room orientation, increasing the realism of the simulation. Finally, an additional input to the model was the introduction of faults to trigger anomalies at specific times.

Using this physical model, the data were generated and labeled with faulty periods of the system. The simulations were initially performed under normal conditions, resulting in a healthy dataset. Subsequent simulations introduced problems of varying lengths that affected the operation of the system. These problems were inserted at random times and alternated between normal and faulty periods.

The dataset obtained after introducing faults comprised three distinct categories. Firstly, fault 1 (F1) was characterized by a fan coil valve that was stuck at a 50% position. In order to accurately simulate this fault, the water flow through the heat exchanger was proportionally reduced according to the position of the stuck valve. Secondly, fault 2 (F2) represented a situation where the room had a reduced airflow with the fan delivering 50% less air. To simulate F2, the air supply to the room was directly reduced by a corresponding amount. Finally, fault 3 (F3), which signified a complete system failure, was simulated by programming the FCU to stop delivering conditioned air to the room. Furthermore, when generating faults, the defined lengths of the normal and faulty periods were selected based on specific ranges. For fault F1, the length of the normal and faulty periods was randomly chosen between 20 and 40 days. Fault F2 had normal and faulty periods of 10 to 30 days and 10 to 40 days, respectively. Fault F3, which occurs less frequently, had a normal period of 5 to 15 days and a faulty period of 1 to 10 days. The inserted faults significantly impacted the room’s air temperature (Room_temp) and the fan speeds (FS_0 to FS_3).

The duration of the periods left enough time for faults to occur, as faults are not always detected immediately. To identify faulty periods, a comparison was made between the healthy and faulty datasets using the air temperature (Room_temp). Fault periods were marked using large deviations in air temperature, as certain conditions had to be met for faults to be detected. For example, faults could only be detected when the FCU system was operating at an adequate capacity. Therefore, periods with significant air temperature deviations were marked as true fault periods, i.e., periods in which faults occurred during the simulation. However, it is important to emphasize that fault F1 is the most subtle since it does not affect the deviations in the temperatures as much as others do.

In the subsequent sections of this paper, we utilize specific terms to refer to the datasets and models associated with each fault. For instance, we refer to the dataset and models involving the first fault as “F1 dataset”/“F1 models”. Similarly, datasets and models concerning faults F2 and F3 are denoted as “F2 dataset”/“F2 models” and “F3 dataset”/“F3 models”, respectively.

### 2.3. Data Preprocessing

In order to optimize the efficiency of fan coil units, it is crucial to address fault detection by integrating DL models into the corresponding systems. However, for a successful integration, it is imperative that the sensors and sensor data exhibit a good quality. Consequently, preprocessing plays a pivotal role in obtaining reliable modeling results, making it the most crucial aspect of the model development pipeline.

First, the data were cleaned of possible errors or outliers, e.g., temperatures equal to zero and “NaN” (not a number) values. Furthermore, the main drawback of working with noisy HVAC system data is the imbalance of classes and the labeling of the data, especially when it comes to predicting binary classes. To overcome these challenges, we used undersampling as a preprocessing step for each dataset, which effectively mitigates the effects of class imbalance and improves overall data quality. The final output consisted of 50% anomalous data and 50% normal data. Although undersampling, as seen here, is a quick and easy way to resolve class imbalance [34], it can lead to information loss if not used properly. However, in our particular scenario where we were dealing with highly imbalanced big data and temperature transitions occurred slowly, this preprocessing step proved extremely beneficial. It not only reduced the computational cost associated with running the model but also produced better performing models.

After the data preparation, we performed preprocessing utilizing two different approaches, depending on whether DL models or RF models were targeted. In the first approach, we prioritized data consistency and result integrity by initially standardizing the data for DL models. Then, we used sliding-window preprocessing that had a defined window size of 144 rows (equivalent to 12 h) with an equal step size. We also established a threshold for error detection, whereby a window was flagged as faulty if it contained at least one observed fault within it. By implementing this configuration, we enhanced the sensitivity of our models in detecting faults while simultaneously preserving the temporal relationships between data points.

The preprocessing phase for DL models proceeded smoothly, without encountering any issues during model training. The models were executed on GPUs with the assistance of data loaders/batches. However, when the same procedure was applied to the RF model, challenges arose due to limitations inherent in RFs and the scikit-learn library. These limitations resulted in RAM problems when processing large datasets.

Hence, to optimize the RF models, we employed a second approach, by introducing an additional step. Namely, each data window was compressed into a single row, containing summarized features. For continuous features, the summarized features included the minimum, maximum, mean, summation, and standard deviation. For binary features, the maximum function was employed together with a defined grouping function which identified ordered units of ones and output the total count of such groups within a window. By employing this compression technique, the original window, initially consisting of 144 rows and 21 features, was transformed into a compressed representation—a single vector with 80 features. This compression technique aimed to enhance the model’s efficiency by reducing memory consumption during execution.

In summary, a total of 10,066 samples from the F1 dataset, 24,540 samples from the F2 dataset, and 25,446 samples from the F3 dataset were used in the preprocessing phase. The input data for the DL models were organized into tensors consisting of 1000 batches, containing windows of 144 data points and 21 channels or features. In contrast, the RF models used an approach that resulted in input windows with 144 data points and a total of 80 features. These preprocessing steps and input dimensions provided a solid basis for a valid comparison between the DL and RF models. The final output dimension was binary: 0 for no detected fault or 1 for a detected fault.

### 2.4. Model Development

**Figure 1:**
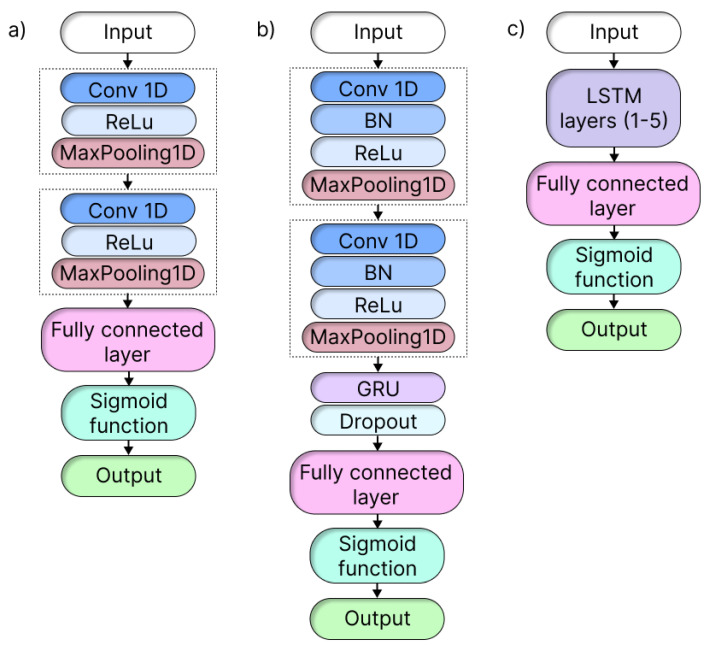
The depicted DL model types of architectures are as follows: (**a**) 1D CNN architecture, comprising two convolutional layers, two ReLu functions, two max-pooling layers, a fully connected layer, and utilizing the sigmoid function for the binary classification; (**b**) 1D CNN+GRU architecture, featuring two convolutional layers, a batch normalization (BN), a ReLU activation, and max pooling layers; additionally, a GRU layer with dropout is incorporated, followed by a fully connected layer utilizing the sigmoid function; (**c**) LSTM architecture, consisting of LSTM layers, a fully connected layer, and utilizing the sigmoid function.

In our effort to develop a comprehensive model, we drew on related work to select the models under consideration. Figure 1 shows the three established architectures that motivated us the most and that made up our model repertoire: 1D CNNs [24,34], CNNs combined with GRU [32], and LSTM [31]. To enable a meaningful comparison between DL models and the traditional approaches, we also included the RF model as a baseline benchmark.

The selected model architectures were chosen for their proven efficiency in FDD tasks. For computational efficiency, accuracy, and feature extraction capability, we chose 1D CNNs. Our 1D CNN architecture included two convolutional layers, two ReLU activation functions, two max-pooling layers, and one fully linked layer [24].

Next, to capture the temporal dependencies in time series datasets, we used LSTM and GRU models. Our LSTM architecture consisted of a single LSTM layer followed by a fully connected layer [31]. We also used a hybrid CNN+GRU architecture that was inspired by previous research [32] and combined the strengths of both components. The CNN component included two convolutional layers, two pooling layers, a batch normalization, and a ReLU activation, while the GRU component was integrated after the last pooling layer and included a dropout and a fully connected layer.

Hyperparameter optimization played a crucial role in maximizing model performance. We used Bayesian optimization with 50 iterations to tune the models using the training and validation datasets. Adam optimization was used for all models. For the DL models, we used the binary cross-entropy loss function and the sigmoid activation function. A detailed description of hyperparameters for the DL models can be found in Table 2. The tuning process for the CNN models focused on optimizing parameters such as kernel size, stride, number of filters, learning rate, and weight decay. Similarly, the CNN+GRU model was optimized with additional parameters such as hidden size and dropout rate. Lastly, the LSTM model involved tuning parameters such as the number of LSTM layers, hidden size, learning rate, and weight decay. Other parameters mentioned in Table 2 such as window size, step size, batch size, activation function, optimizer, cost function, epoch, patience for early stopping, and tuning iterations were all defined with fixed values for each model.

To ensure the integrity of model training, we shuffled the input dataset exclusively for training, thus introducing an additional layer of randomness. However, it is important to note that shuffling was not applied to the validation and testing datasets to maintain impartiality during subsequent evaluations.

In addition to the DL models, we also used the RF model as a baseline, as it has been shown to outperform other approaches in previous research [37]. Namely, RF-based solutions could offer notable advantages that position them as comparable contenders to DL models. These advantages include interpretability, feature importance determination, effective capture of nonlinear relationships, robustness to outliers, insensitivity to feature scaling, and efficiency and scalability when processing datasets.

The RF model was configured using parameters such as the number of trees, maximum depth, maximum features, minimum samples for split, minimum samples for leaf, maximum features, and criterion. The specific ranges of values for these parameters are also defined in Table 2.

## 3. Results and Discussion

The preprocessing results revealed a significant size reduction in the F1 dataset, primarily due to its initial class imbalance of only 3–4% labeled faults compared to the other datasets. Consequently, the F1 dataset was reduced to approximately 4 to 5 thousand rows. In contrast, the preprocessing techniques produced more favorable outcomes for the F2 dataset (labeled 12–13%) and the F3 dataset (labeled 21–23%), resulting in sizes ranging around 12 thousand rows and 13 thousand rows, respectively.

For the hyperparameter tuning process, the outcomes of the DL and RF models tuning are summarized in Table 3.

Upon examining the summarized results, several key observations can be made. Starting with the confusion matrices presented in Table 4, we can observe the performance of the models in terms of true negatives, false positives, false negatives, and true positives for each fault category. For F1 models, both the CNN and CNN+GRU models achieved similar results, with around 44% true negatives, 5% false positives, 5% false negatives, and 45% true positives. In contrast, the LSTM and RF models exhibited weaker results.

Moving on to the F2 models, the CNN+GRU model exhibited the highest accuracy among all models, with around 46% true positives. The CNN, LSTM, and RF models achieved relatively similar results, with varying distributions of the confusion matrix indicators.

For the F3 models, both the CNN+GRU and CNN models showcased strong performance, with approximately 47% true negatives and true positives. The LSTM and RF models demonstrated slightly lower performance, with different trade-offs between true negatives, false positives, false negatives, and true positives.

Next, Table 5 presents summarized metrics for each fault model, including accuracy, precision, recall, $F_{1}$ score, and area under the curve (AUC). For F1 models, the CNN+GRU model attained the highest accuracy of 90% and an $F_{1}$ score of 0.91, indicating a favorable balance between precision and recall. The RF model yielded satisfactory results with an accuracy of 84% and an $F_{1}$ score of 0.85, outperforming the LSTM model which exhibited comparatively lower performance. This outcome can be attributed to data scarcity, as the models from DL usually cope better with larger datasets and therefore provide more accurate results in such a context.

In the case of the F2 models, the CNN+GRU model showcased the highest accuracy of 93% and an $F_{1}$ score of 0.93, indicating strong overall performance. The RF model performed the weakest among all models, while the LSTM and CNN models demonstrated similar results with accuracy ranging from 0.89 to 0.90.

Regarding the F3 models, both the CNN+GRU and CNN models achieved high accuracy scores of 93% and 95%, respectively, showcasing robust performance. The LSTM model displayed a slightly lower accuracy of 0.89, while the RF model exhibited the weakest performance with an accuracy of 0.85.

**Figure 2:**
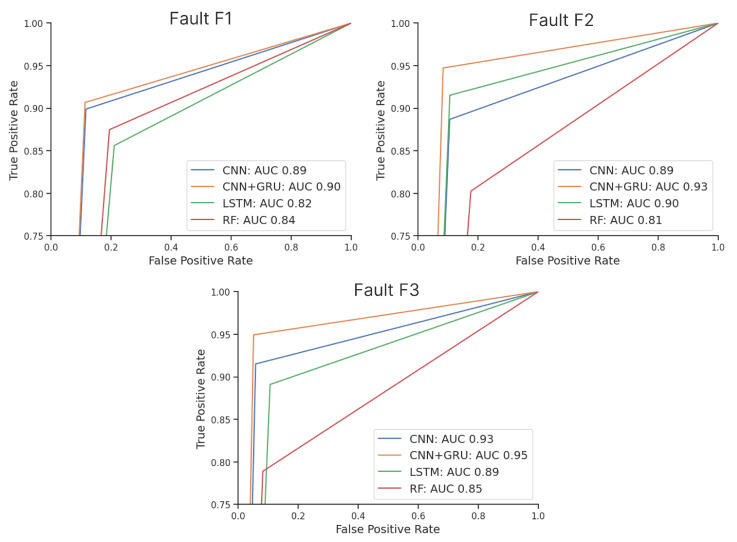
ROC curves and AUC scores calculated on the test subset for each model and each fault.

Furthermore, Figure 2 presents a visual comparison of the receiver operating characteristic (ROC) curves, which effectively highlight the distinctions in model performance and allow for some new interpretations. For example, the utilization of undersampling as a preprocessing step resulted in the F1 dataset being the smallest, followed by the F2 and F3 datasets (with the F3 dataset being the largest). While maintaining a balanced distribution of 50% faults and 50% nonfaulty data, the dataset sizes played a significant role in determining the final results. Figure 2 clearly demonstrates that the curves for the F1 models are closer together compared to those of the F2 and F3 models, indicating that the smaller dataset led to lower performance across all models.

Upon analyzing the F2 and F3 models, it becomes evident that as the dataset size increases, the model curves diverge. Notably, the RF model’s performance deteriorates, suggesting that larger dataset sizes provide more benefit to DL models while adversely affecting traditional models. However, despite DL models exhibiting poorer performance with less data, the CNN+GRU consistently outperformed each individual model, regardless of the dataset size. This finding highlights the robustness of the hybrid model in accommodating varying dataset sizes.

**Figure 3:**
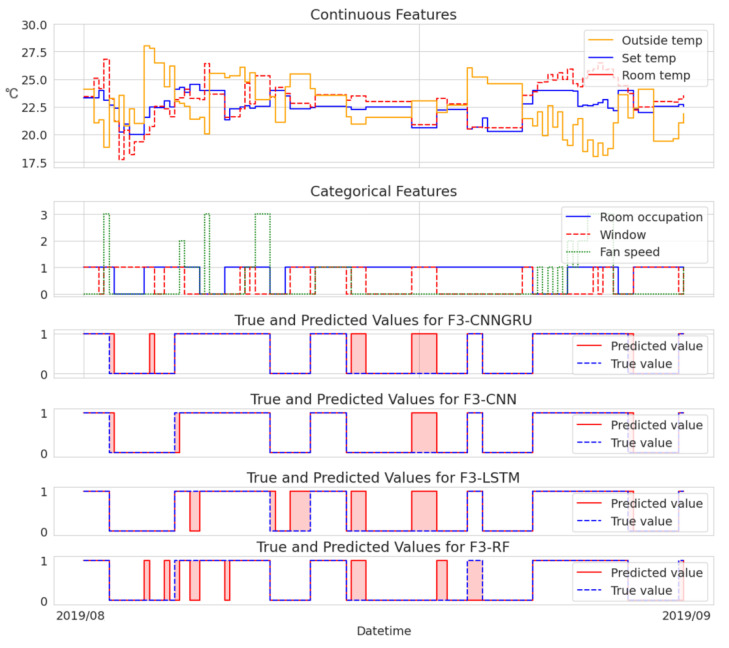
Example of sensitive fault detection for the F3 dataset.

**Figure 4:**
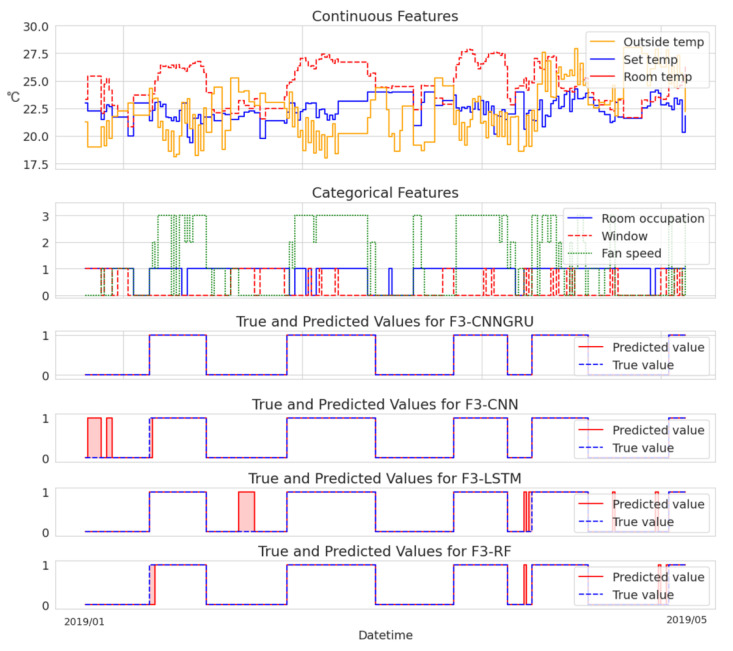
Accurate F3 fault detection by the CNN+GRU model, outperforming other models.

To provide an analysis of the variations in model performance, we additionally present two cases visualized in Figure 3 and Figure 4. These cases allow for a direct comparison of each model’s fault detection capabilities.

Figure 3 provides visual representations of instances where the models exhibited incorrect detections, offering valuable insights into their limitations and areas for improvement. This analysis sheds light on the weaknesses of each model, particularly in relation to the sensitivity of the threshold for anomaly labeling.

In contrast, Figure 4 highlights examples where the CNN+GRU model excelled in terms of accurate detections compared to the other models. These instances serve as compelling evidence of the superior performance and effectiveness of the CNN+GRU model. While the other models also demonstrate satisfactory detection of obvious temperature deviations depicted in the graph, they tend to yield more false positives by detecting faults that do not exist within the selected data period. This tendency can be attributed, in part, to the sensitivity of the fault-labeling mechanism.

Overall, the presented cases play an important role in conveying the strengths and weaknesses of the models, allowing for a comprehensive understanding of their performance in fault detection.

It is crucial to emphasize that any gaps in the data resulting from the data preprocessing stage were disregarded for the purpose of visualization. The selected continuous features included outside temperature, set temperature, and room temperature, while the categorical features encompassed room occupation, window status, and fan speed.

## 4. Conclusions

In this paper, an originally curated dataset was labeled by simulating three different faults within a physical model implemented in TRNSYS. To prepare the data for analysis, different preprocessing techniques were applied, such as undersampling with sliding windows for DL models and with an additional compression of the sliding windows for RF models. Among the four models tested (CNN, CNN+GRU, LSTM, RF), the hybrid CNN+GRU model performed better than the others for each fault. This finding suggests the possible superiority of combining different model architectures.

This research makes a contribution to the field of fault detection in HVAC systems by using a larger and more diverse dataset. The dataset included a combination of real-world data (originated from a hotel’s sensory measurement system, additionally augmented with environmental conditions data) and simulated faults.

One of the key findings of this study is the remarkable performance of the hybrid CNN+GRU model. Regardless of the size of the dataset, this model showed robustness and outperformed the other models. In addition, the models from the DL family generally performed better than the traditional baseline model, highlighting the effectiveness of deep learning in fault detection.

The superior performance of the CNN-based models can be attributed to their ability to effectively capture spatial and temporal patterns in the input data, which enables the extraction of meaningful features for fault detection in FCU systems. The inclusion of a GRU further improves the performance of the model by capturing long-term dependencies and temporal dynamics.

In contrast, despite its widespread use in sequence modeling, the LSTM model did not perform as well as the CNN-based models. This suggests that the FCU fault detection problem is primarily based on the detection of local patterns and faults, which suits the strengths of the CNN models. Moreover, the optimization process for the LSTM model proved to be more difficult due to its longer convergence time compared to other models. The successful implementation of LSTM requires a more complex network structure.

The traditional baseline RF model showed relatively lower performance compared to the DL models, suggesting that the DL models’ ability to automatically learn hierarchical representations better captured the complex patterns and relationships in the FCU data.

In conclusion, the application of DL models, especially hybrid architectures such as CNN+GRU, holds great promise for improving fault detection and diagnosis in HVAC/FCU systems. By accurately identifying and diagnosing faults, DL models have the potential to optimize system performance, reduce maintenance costs, and extend the life of HVAC systems. However, to assess the generalizability and robustness of the proposed models, further validation using real data from FCU systems in actual hotel buildings is required. Such validation would provide insights into real-world scenarios and support decision-making in the development of fault detection systems.

## Figures and Tables

**Table 1:** Description and data types of dataset features.

Feature	Description	Data Type
Datetime	Date and time.	datetime
Hvac_mode	1 = FCU heating/0 = FCU cooling.	binary
Hvac_state	1 = FCU on/0 = FCU off.	binary
Room_occupancy	1 = room occupied/0 = room unoccupied.	binary
Window	1 = window open/0 = window closed.	binary
Hvac_state_manual	1 = HVAC control guest-regulated.	binary
FS_0	1 = fan turned off.	binary
FS_1	1 = first speed.	binary
FS_2	1 = second speed.	binary
FS_3	1 = third speed.	binary
Orientation_S	1 = south room orientation.	binary
Orientation_W	1 = west room orientation.	binary
Orientation_N	1 = north room orientation.	binary
Orientation_E	1 = east room orientation.	binary
Set_temp	Set temperature of a room (${}^{\circ }$C).	int
Room_temp	Measured room temperature (${}^{\circ }$C).	int
Room_temp_up	Measured temp. of room upstairs (${}^{\circ }$C).	int
Room_temp_down	Measured temp. of room downstairs (${}^{\circ }$C).	int
Room_temp_cw	Measured temp. of right-neighboring room (${}^{\circ }$C).	int
Room_temp_ccw	Measured temp. of left-neighboring room (${}^{\circ }$C).	int
Outside_temp	Outside air temperatures (${}^{\circ }$C).	float
Irradiation	Solar energy reaching a surface (W/m${}^{2}$).	float

**Table 2:** Hyperparameter values that were used in our experiments for Bayesian optimization of all models. These values were set based on reported related work and on our prior experience.

Hyperparameters	Values
**CNN, CNN+GRU, and LSTM**	
Learning rate	0.0001–0.1
Weight decay	0.0001–0.1
Batch size	1000
Optimizer	Adam
Cost function	Binary cross-entropy loss
Epoch	30
Patience for early stopping	5
** CNN specific**	
Kernel size	2–3
Stride	2–3
Number of filters	36–128
** CNN+GRU specific**	
Kernel size	2–3
Stride	2–3
Number of filters	36–128
Hidden size	64–128
Dropout rate	0.0–0.5
** LSTM specific**	
Number of LSTM layers	1–5
Hidden size	64–128
**RF**	
Number of estimators	100–1000
Maximum depth	3–20
Maximum features	sqrt, log2
Minimum samples split	2–100
Minimum samples leaf	1–10
Criterion	gini, entropy
**All models**	
Window size	144 rows (12 h)
Step size	144 rows (12 h)
Threshold value	1 (5 min)
Tuning iterations	50

**Table 3:** Varying hyperparameter values of the best-performing models, obtained through Bayesian optimization. Hyperparameter values we explored are shown in Table 2.

Model-Fault	Learning Rate	Weight Decay	Kernel Size	Number of Filters	Stride	Hidden Size	Dropout	LSTM Layers
CNN-F1	0.1	0.0009	2	93	3	-	-	-
CNN-F2	0.02	0.0001	2	104	3	-	-	-
CNN-F3	0.001	0.0001	3	128	3	-	-	-
CNN+GRU-F1	0.007	0.0001	2	109	2	90	0.32	-
CNN+GRU-F2	0.015	0.0001	2	36	2	71	0.5	-
CNN+GRU-F3	0.01	0.0001	2	128	3	64	0.5	-
LSTM-F1	0.017	0.002	-	-	-	64	-	1
LSTM-F2	0.04	0.0001	-	-	-	64	-	1
LSTM-F3	0.02	0.0001	-	-	-	66	-	2
	**Number of Trees**	**Max. Depth**	**Max. Features**	**Minimum Samp. Split**	**Minimum Samp. Leaf**	**Criterion**		
RF-F1	1000	17	sqrt	2	1	gini		
RF-F2	885	20	sqrt	2	1	entropy		
RF-F3	1000	20	sqrt	2	1	gini		

**Table 4:** Ratios of true/false positives/negatives for each fault and each model, obtained on the test subset.

Model	True Negatives	False Positives	False Negatives	True Positives
**Fault 1**				
CNN	44.16%	5.84%	5.06%	44.94%
CNN+GRU	44.25%	5.75%	3.84%	46.16%
LSTM	39.45%	10.55%	7.20%	42.80%
RF	40.24%	9.76%	6.26%	43.74%
**Fault 2**				
CNN	44.70%	5.30%	5.66%	44.34%
CNN+GRU	45.79%	4.21%	2.64%	47.36%
LSTM	44.68%	5.32%	4.24%	45.76%
RF	41.17%	8.83%	9.88%	40.12%
**Fault 3**				
CNN	47.06%	2.94%	4.24%	45.76%
CNN+GRU	47.40%	2.60%	2.54%	47.46%
LSTM	44.65%	5.35%	5.45%	44.55%
RF	45.87%	4.13%	10.56%	39.44%

**Table 5:** Model performance results for each fault and each model, obtained on the test subset. The best results are printed in boldface.

Model	Accuracy	Precision	Recall	$F_{1}$	AUC
**Fault 1**					
CNN	0.89	0.89	0.90	0.89	0.89
**CNN+GRU**	**0.90**	**0.89**	**0.92**	**0.91**	**0.90**
LSTM	0.82	0.80	0.86	0.83	0.82
RF	0.84	0.82	0.87	0.85	0.84
**Fault 2**					
CNN	0.89	0.89	0.89	0.89	0.89
**CNN+GRU**	**0.93**	**0.92**	**0.95**	**0.93**	**0.93**
LSTM	0.90	0.90	0.92	0.91	0.90
RF	0.81	0.82	0.80	0.81	0.81
**Fault 3**					
CNN	0.93	0.94	0.92	0.93	0.93
**CNN+GRU**	**0.95**	**0.95**	**0.95**	**0.95**	**0.95**
LSTM	0.89	0.89	0.90	0.89	0.89
RF	0.85	0.91	0.79	0.84	0.85

## Data Availability

Not applicable.

## Abbreviations

The following abbreviations are used in this manuscript:

HVAC	Heating, ventilation, and air conditioning
FCUs	Fan coil units
FD	Fault detection
DL	Deep learning
CNN	Convolutional neural network
GRU	Gated recurrent unit
LSTM	Long short-term memory network
RF	Random forest
IEA	The International Energy Agency
BN	Batch normalization
